# Pollution-driven viral susceptibility: mixed contaminants as hidden determinants of aquaculture disease

**DOI:** 10.1007/s44307-025-00091-7

**Published:** 2025-12-09

**Authors:** Shaoping Weng, Liqing Wu, Changjun Guo

**Affiliations:** State Key Laboratory of Biocontrol, Southern Marine Science and Engineering Guangdong Laboratory (Zhuhai), China-ASEAN Belt and Road Joint Laboratory on Mariculture Technology, Specialized Laboratory of Aquatic Animal Disease, School of Life Sciences, Sun Yat-sen University, Guangzhou, 510275 China

Viral contamination in aquatic environments is increasingly recognized as a risk not only to farmed animals but also to human health through seafood safety, food-borne exposure, and disruption of coastal ecosystems (Li et al. 2024). Microplastics (MPs) and bisphenol A (BPA), which are now ubiquitous in marine and freshwater systems, can carry pathogens, concentrate co-pollutants, and interfere with immune and endocrine regulation along the food web, raising concern from a One Health perspective (Gao et al. 2025). Against this broader backdrop of pollution-driven vulnerability, viral outbreaks in aquaculture have traditionally been attributed to pathogen virulence, host genetics and farming intensification, yet growing evidence suggests that disease emergence often arises not from stronger viruses but from contaminated environments that suppress host defenses. In this context, MPs and BPA are no longer merely toxic chemicals, they function as non-traditional ecological drivers that shape viral susceptibility in farmed fish and invertebrates, repositioning aquatic virology within an environmentally mediated framework and establishing pollutant exposure as a central determinant of disease risk.

## Pollution-mediated viral reservoirs in aquaculture

MPs in aquatic systems are no longer passive debris but active biological interfaces. By supporting Plastisphere biofilms loaded with *Vibrio* and antimicrobial resistant bacteria, they can stabilize viral particles, modify waterborne transmission and act as non-host reservoirs and co-transporters of microbial hazards (Pavi et al. 2025; Shan et al. 2023). In many coastal farming areas, aquaculture operates in waters already affected by diffuse plastic pollution from urban, industrial, and riverine inputs, while plastic-based ropes, nets, buoys, cages and feeding devices further add to the MP burden through gradual weathering and abrasion, generating particles that can be ingested and retained by cultured species (Bebianno et al. 2025; Eliso et al. 2025). At the same time, MPs interact with antibiotics and heavy metals to create multi-stress environments and favour resistance development in surrounding waters (Sun et al. 2025). Thus, in polluted farming environments, aquatic viruses do not merely encounter susceptible hosts, they increasingly encounter plastic platforms that stabilize, concentrate, and disperse them, potentially amplifying transmission opportunities.

## Mixed contaminants reprogram immune baselines rather than induce acute toxicity

The most consequential effects of MPs and BPA are not lethal toxicity but physiological reprogramming that lowers the threshold for viral infection. Across teleost models, dietary exposure to polyvinyl chloride (PVC), polyethylene (PE) or polypropylene (PP) MPs has been shown to induce histopathological lesions, redox imbalance and immunoregulation disturbances, suppress antiviral responses and increase viral replication in immune organs, indicating that plastic particles can markedly alter the basal physiological status of fish (Espinosa et al. 2019, Li et al. 2021). Recent work in largemouth bass further demonstrates that environmentally realistic co-exposure to MPs and BPA inhibits NRF2-driven antioxidant signaling, reduces hepatic SOD1, CAT and GPx activities, elevates ROS and lipid peroxidation, disrupts mitochondrial function, and depletes ATP, ultimately activating caspase-dependent apoptosis and creating a biochemical milieu that favours nervous necrosis virus (NNV) replication (Gao et al. 2025; Huang et al. 2024; Zheng et al. 2025).

**Fig. 1:**
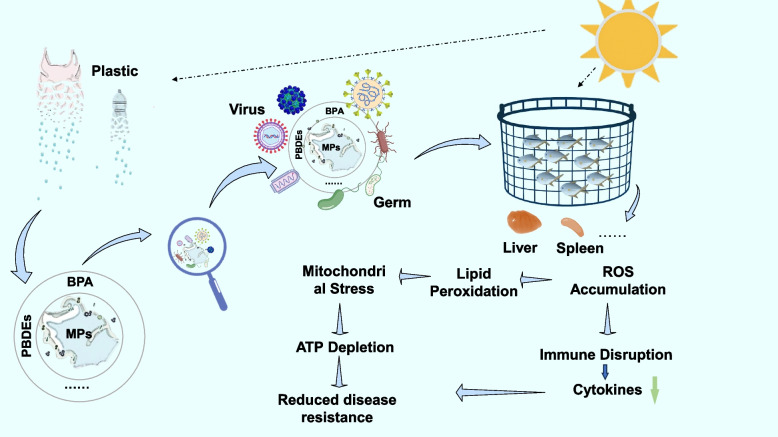
Mixed contaminants lower disease resistance in farmed fish. MPs and co-leached chemicals such as BPA and PBDEs enter aquaculture waters from multiple sources, forming contaminated particles that can adsorb viruses and microbes. After uptake by fish, these mixtures induce mitochondrial stress, ROS accumulation and lipid peroxidation, leading to ATP depletion, cytokine dysregulation, and broader immune disruption. The resulting reduction in disease resistance lowers the threshold for successful viral and opportunistic microbial infections in polluted farming environments

Mixed pollution amplifies these effects beyond MPs alone. In crustaceans and fish, combinations of MPs with dissolved pollutants disrupt innate immune signaling and gut microbiota, while hydrophobic contaminants such as polybrominated diphenyl ethers (PBDEs) “hitchhike” on MPs, accumulate in tissues and initiate ferroptosis-related immune impairment (Rios-Fuster et al. 2022, Li et al. 2022; Chen et al. 2024). At the environmental interface, MP–BPA interactions are governed by surface aging, functionalization, and water chemistry, which influence sorption–desorption dynamics and bioavailability, and BPA in coastal aquaculture waters undergoes salinity- and dissolved organic matter (DOM)- dependent phototransformation that generates additional reactive metabolites (Jeon et al. 2024, Beatriz et al. 2024). Consistent with these mechanisms, combined polystyrene MPs and BPA exposure alters oxidative and metabolic biomarkers in embryos (Eliso et al. 2025). Collectively, these studies demonstrate that mixed contaminants reprogram host redox and immune baselines through ROS accumulation, compromised antioxidant defence, and mitochondrial dysfunction, ultimately reducing disease resistance and elevating viral susceptibility (Fig. 1).

## Microplastic pollution threatens viral control in aquaculture

Aquaculture systems are increasingly challenged by the accumulation of microplastics, BPA and other co-contaminants in surrounding coastal waters. These particles and chemicals can enter farms through seawater intake, riverine inputs, resuspension of legacy plastics from sediments and the use of contaminated feeds or additives. Once present in culture units, MPs and associated pollutants interact with dissolved organic matter and microbiota, favouring the formation of Plastisphere biofilms that host bacteria and adsorb viruses. For farmed fish and shellfish, this means that routine exposure to rearing water may be accompanied by chronic intake of MPs, contaminant, pathogen complexes that induce subclinical immunosuppression. Therefore, viral susceptibility emerges because of environmental pollution pressure acting on cultured animals, rather than an intrinsic weakness of aquaculture itself.

Recognizing that mixed pollutants can erode the antiviral capacity of stock highlights an additional layer of risk that overlays traditional biosecurity concerns. Even when vaccines, husbandry and genetic selection are optimized, elevated burdens of MPs and BPA in the farming environment may lower the threshold for viral infection by driving oxidative stress, disrupting immune signaling and altering host–microbiota relationships. Integrating pollution monitoring and control into farm management therefore becomes an important complement to classical disease control. Implementing measures such as improving water quality protection in surrounding catchments, reducing external plastic inputs, selecting less contaminant prone materials, and augmenting antioxidant and immune defenses in cultured species can all help to mitigate the impact of environmental pollution.

Taken together, these observations indicate that microplastic- and BPA-driven susceptibility represents an emerging challenge for otherwise sustainable aquaculture systems. Integrating strategies to mitigate contaminant sources and reduce their bioaccumulation with measures to support the antioxidant and immune competence of cultured species will therefore complement conventional strategies based on selective breeding, vaccination, and improved husbandry (Xu et al. 2024).
